# Supplementary material for: MutL sliding clamps coordinate exonuclease-independent Escherichia coli mismatch repair
Source: Nat Commun. 2019 Nov 22;10:5294. doi: 10.1038/s41467-019-13191-5 (PMC6876574; doi:10.1038/s41467-019-13191-5)
Supplement: Supplementary file 1 — Supplementary Information [file 41467_2019_13191_MOESM1_ESM.pdf]

Supplementary Information For

MutL Sliding Clamps Coordinate Exonuclease-Independent

Escherichia coli Mismatch Repair

Liu et al.,

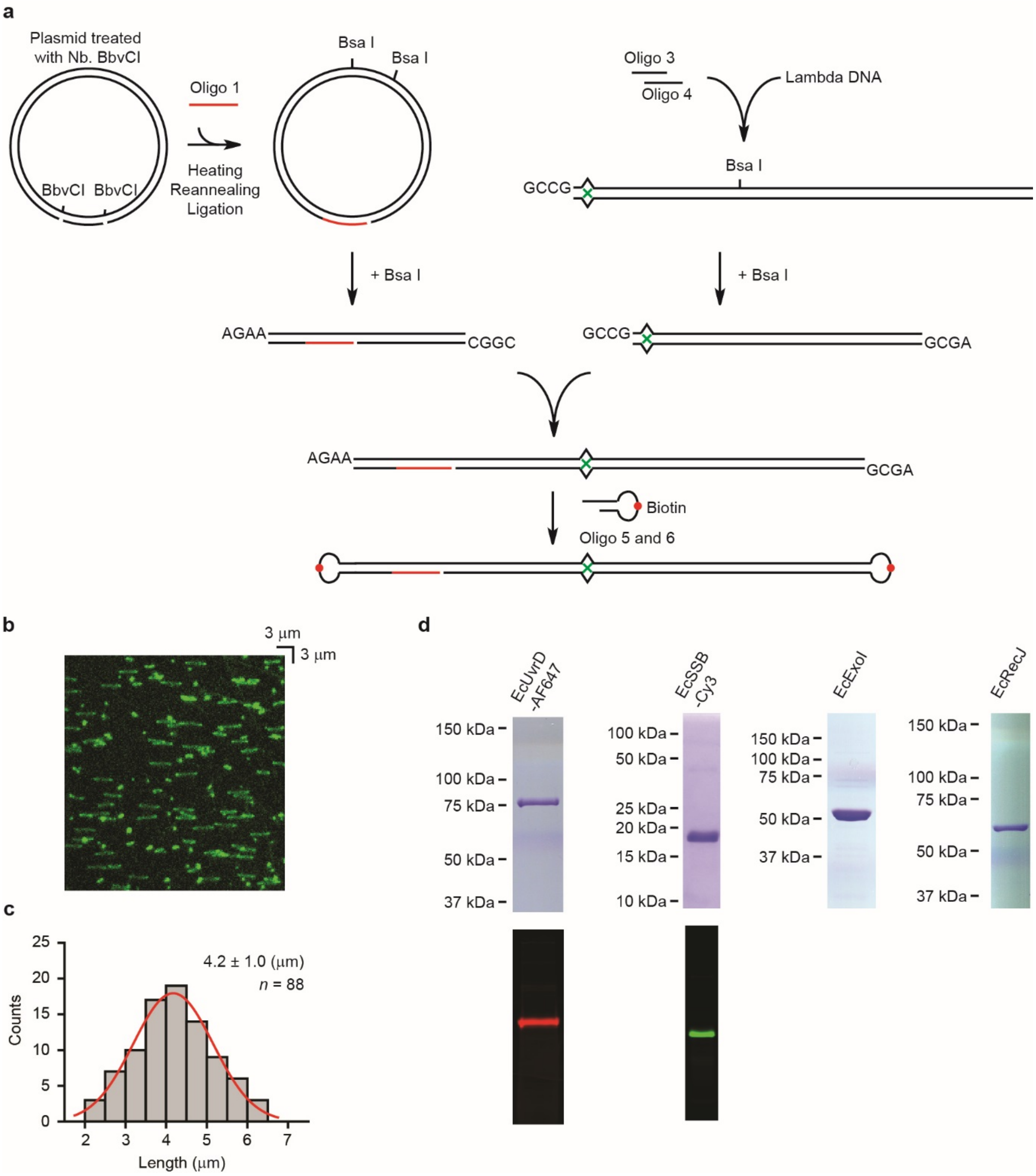

Supplementary Figure 1. Construction of Mismatched DNA and Purification of *E. coli* MMR Proteins. (**a**) An illustration of the construction protocol for the 18.4-kb mismatched DNA containing a single strand break and helicase-resistant self-annealing ends (see **Methods**). (**b**) Representative mismatched DNAs visualized by smTIRF microscopy in the absence of flow.

The DNA was stained with Sytox Orange. A 40 x 40 μm field of view is shown. (**c**) The length distribution of the mismatched DNA observed by smTIRF microscopy. The data was fit with a Gaussian distribution that determined the mean ± s.d. (**d**) Coomassie stained (top) and fluorescent (bottom) images of SDS-PAGE gels showing the labeled and unlabeled MMR proteins.

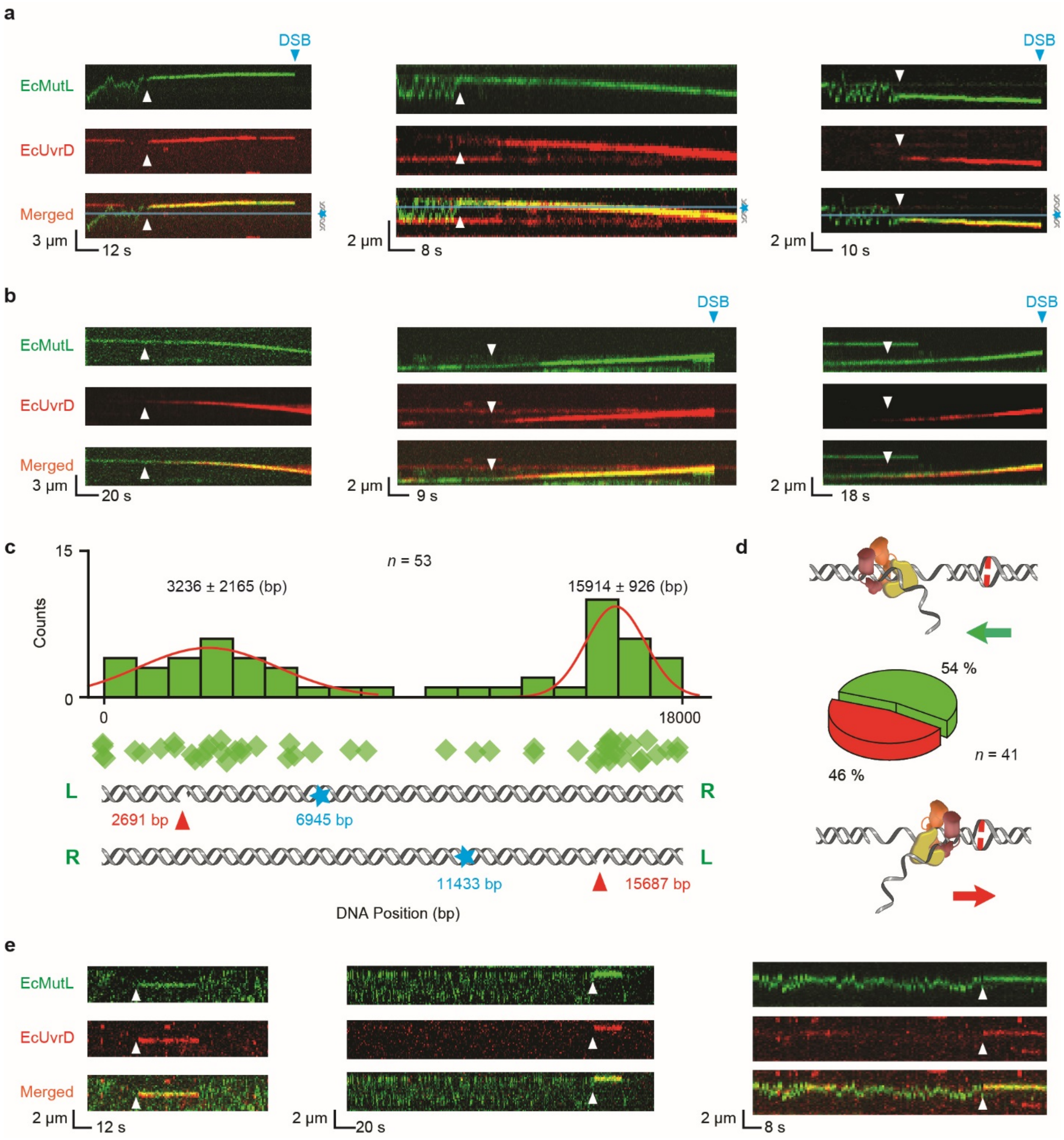

Supplementary Figure 2. Representative Kymographs of EcMutL-EcUvrD Complex. (**a**) Representative kymographs showing that the AF555-EcMutL(green)-AF647-EcUvrD(red) complex unwinds DNA. White arrowheads indicate association of fast-diffusing EcMutL sliding clamp with EcUvrD; Blue arrowheads indicate the occurrence of a DSB. Blue line and star indicate the mismatch position. (**b**) Representative kymographs showing that the

(unlabeled)EcMutS-(AF555)EcMutL-(AF647)EcUvrD complex unwinds DNA. White arrowheads indicate association of slow-diffusing EcMutS-EcMutL complex with EcUvrD; Blue arrowheads indicate the occurrence of a DSB. (**c**) The distribution of starting positions for EcMutL-EcUvrD complex on DNA. The two possible orientations of the mismatched DNA with strand break position (Blue stars: mismatch; red arrowheads: strand break). Diamonds represent individual starting event. The data was fit with a Gaussian distribution that determined the mean ± s.d. (**d**) A schematic illustration and frequency of EcMutL-EcUvrD complex directional unwinding (red: toward the mismatch; green: away from the mismatch). (**e**) Representative kymographs showing the formation of EcMutL-EcUvrD complex in the presence of EcSSB (200 nM). Arrowheads indicate association of EcMutL sliding clamp with EcUvrD.

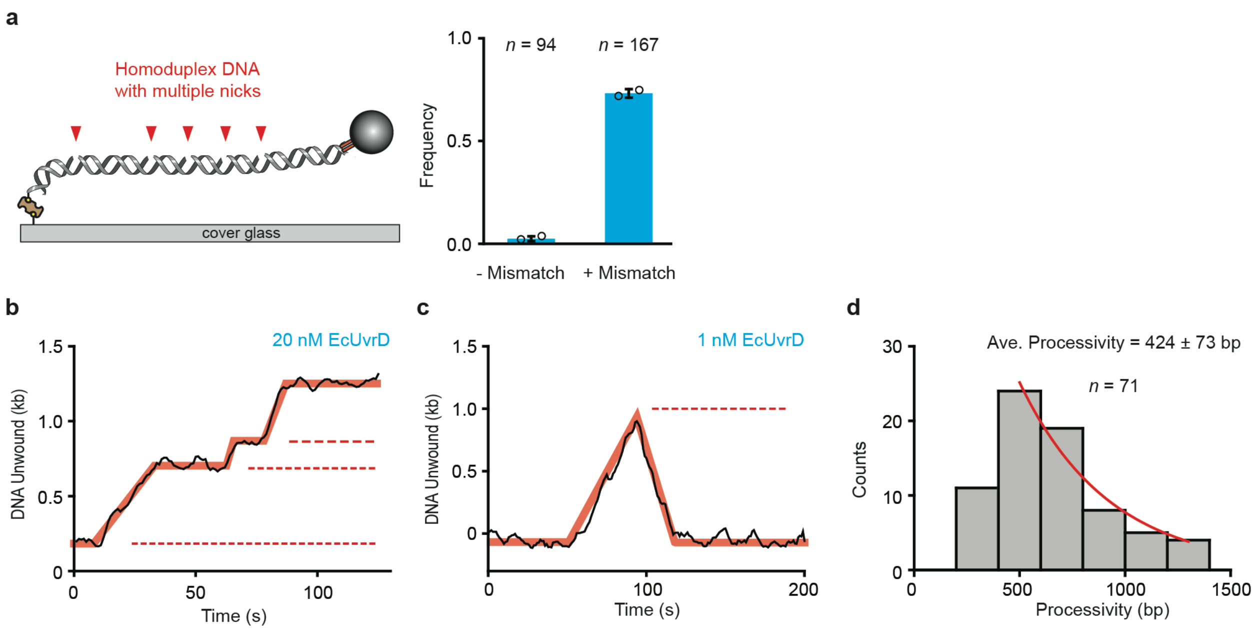

Supplementary Figure 3. Frequency and Processivity Analysis. (**a**) An illustration of the smFS homoduplex (no mismatch) DNA substrate containing multiple strand breaks (left). The frequency of unwinding with and without a mismatch in the presence of EcMutS (100 nM), EcMutL (100 nM), EcUvrD (20 nM) (right, mean ± s.d.; n = number of DNA molecules). Open circles represent the frequencies from separate experiments at the indicated conditions. (**b**) Representative time trajectories of the SPM beads showing individual unwinding step at EcMutS (100 nM), EcMutL (100 nM) and EcUvrD (20 nM). Red dotted lines indicate the pause/dissociation of EcUvrD between multiple steps. (**c**) Representative time trajectories of the SPM beads showing individual unwinding step at EcMutS (100 nM), EcMutL (100 nM) and EcUvrD (1 nM). Red dotted line indicates pause/dissociation of EcUvrD. (**d**) Histogram of binned unwinding processivity of EcMutS (100 nM), EcMutL (100 nM) and 1 nM EcUvrD (1 nM) that were fit to a single exponential decay to derive the average processivity (mean ± s.e.; n = number of events).

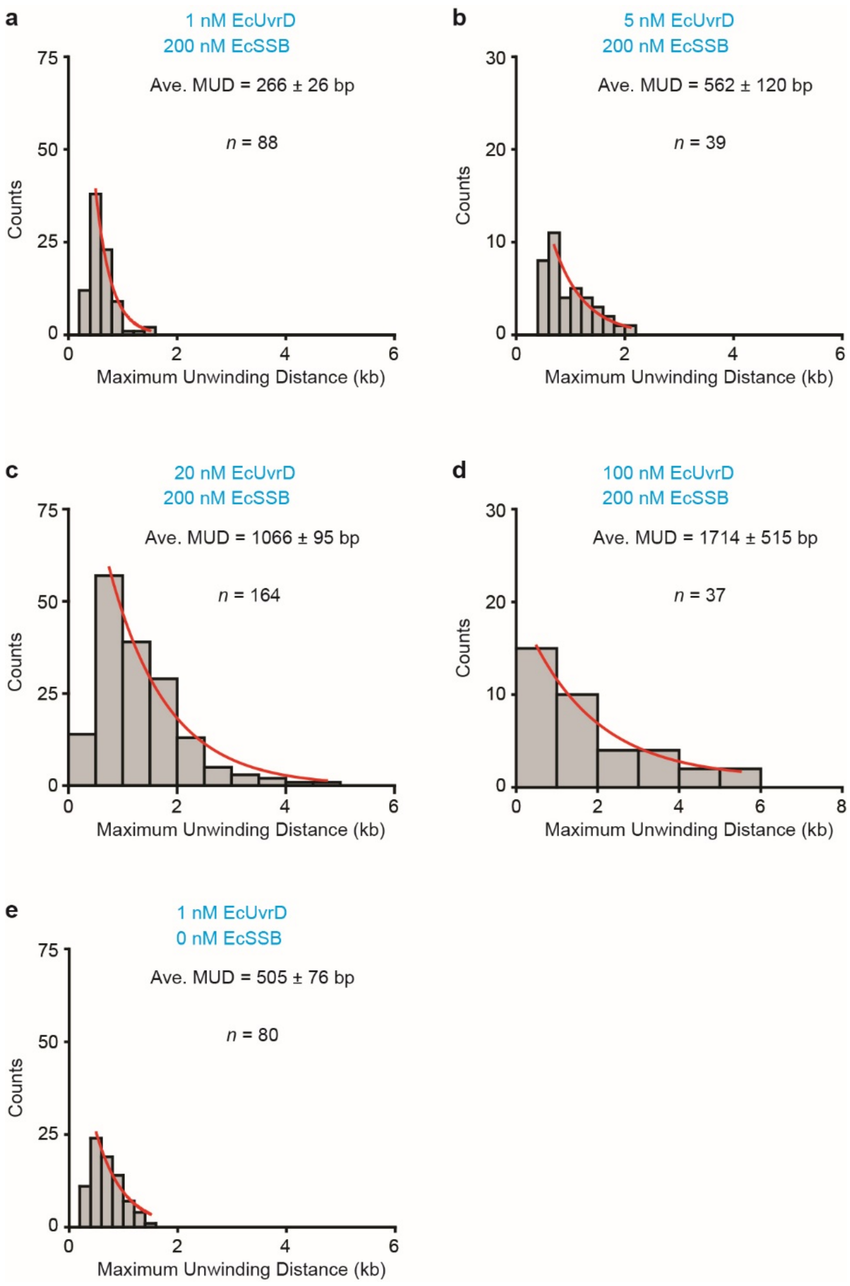

Supplementary Figure 4. Histogram of Binned Maximum Unwinding Distance of EcMutL-tethered EcUvrD. Maximum unwinding distances (MUD) at various conditions were fit to a single exponential decay to derive the average length (mean ± s.e.; n = number of events). Experiments were performed in the presence of EcMutS (100 nM), EcMutL (100 nM) and various concentrations of EcUvrD and EcSSB.

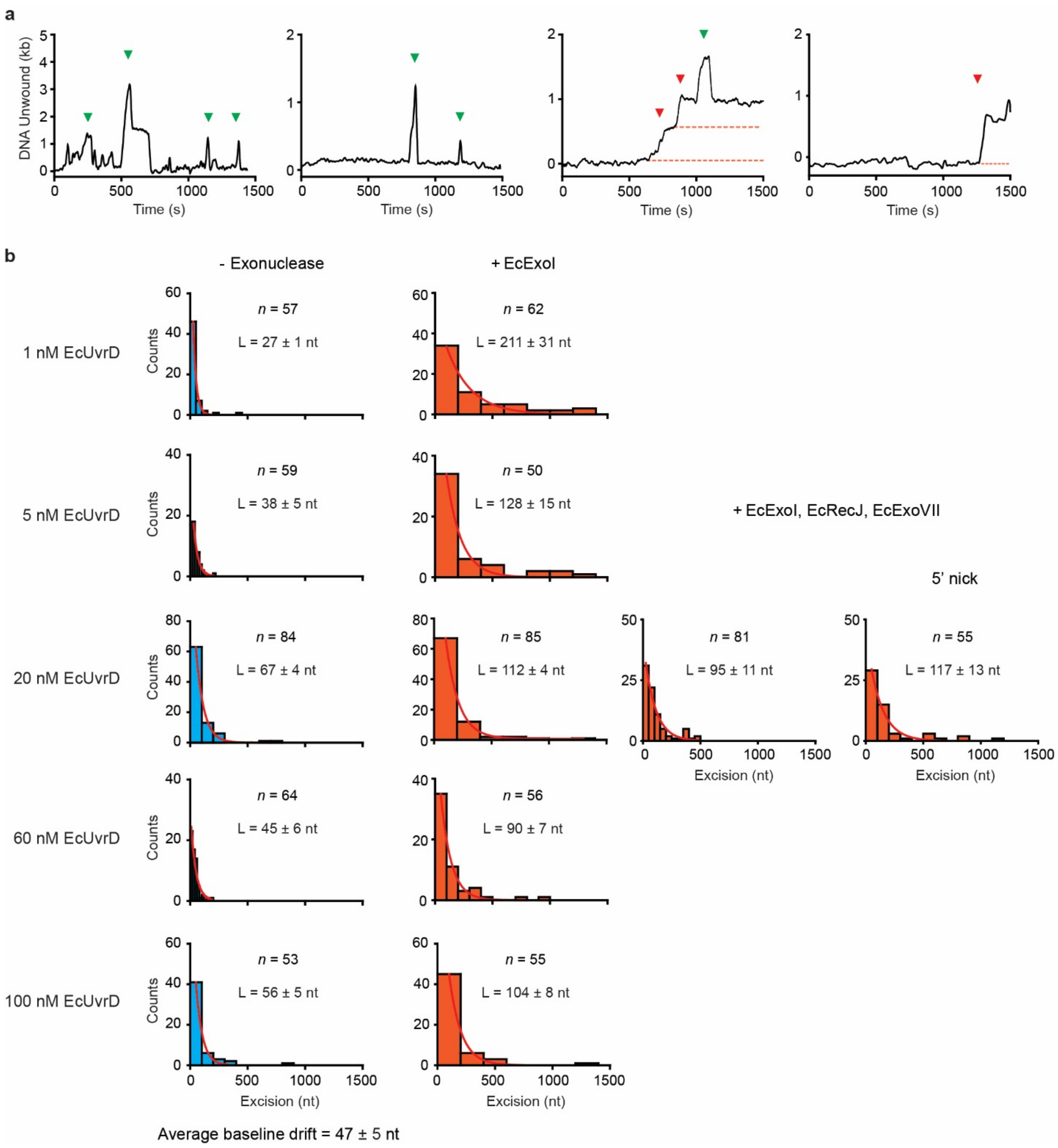

Supplementary Figure 5. Representative Trajectories and Histogram of Binned Excision Length. (**a**) Representative time trajectories of the SPM beads in the presence of EcMutS (100 nM), EcMutL (100 nM), EcUvrD (20 nM), EcSSB (200 nM) and EcExoI (20 nM) (see **Methods**). The unwinding followed by rezipping are indicated either as non-excision events (green

arrowheads) or excision events (red arrowheads). The baseline of each excision event is indicated by a red dotted line. (**b**) Excision length (see **Methods**) at various conditions were fit to a single exponential decay to derive the average length (mean ± s.e.; n = number of events).

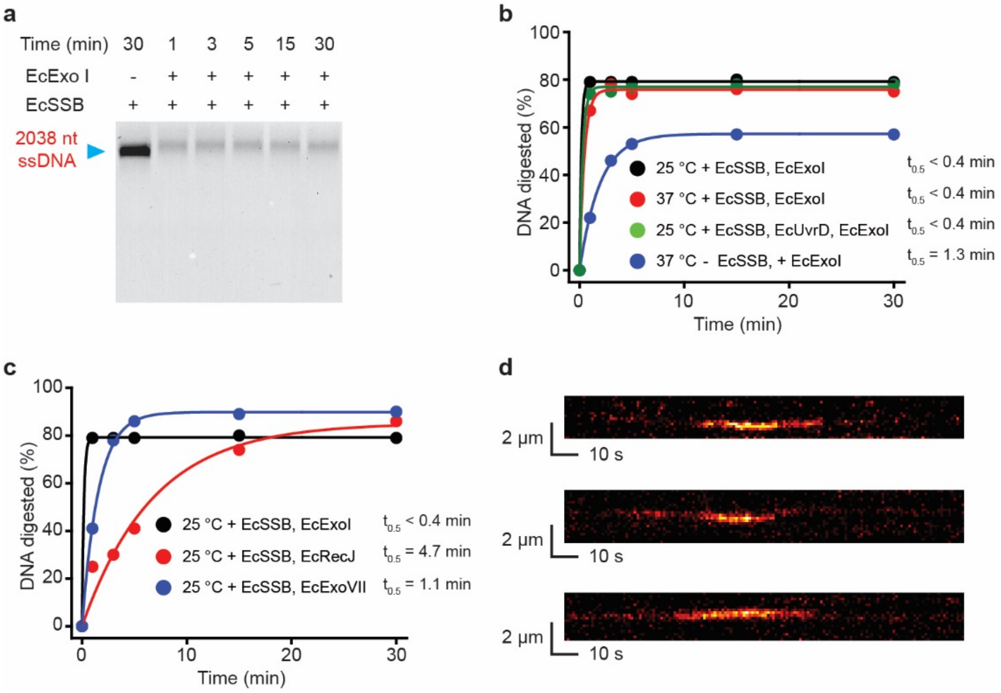

Supplementary Figure 6. ssDNA Exonuclease Activity Assays and Non-Excision Kymographs during MMR. Representative gel (**a**) and quantifications (**b**,**c**) of ssDNA exonuclease activity at various conditions. Data were fit by single exponential equation to derive the half digestion time ($t_{0.5}$). (**d**) Representative kymographs of Cy3-EcSSB during MMR. The Cy3-EcSSB binding followed by complete dissociation indicated the absence of excision. Data were acquired by smTIRF in the presence of EcMutS (100 nM), EcMutL (100 nM), EcUvrD (20 nM), EcSSB (200 nM) and all three ssDNA exonucleases (see **Methods**).

Supplementary Table 1. Oligonucleotides

Name	Sequences
Oligo 1	TGAGGGATCGGATGC
Oligo 2	TCAGCATCCGATCCC
Oligo 3	Phos-AGGTCGTCGCCCAGAAGATGGGCGAGTTTG
Oligo 4	Phos-GCCGCAAACTCGCCCATCTTCT
Oligo 5	Phos-TTCTTGGAGTCCACTGCAGTT/Biotin-T/TCTGCAGTGGACTCCA
Oligo 6	Phos-CGCTTAGTGCTATGATGCGTT/Biotin-T/TCGCATCATAGCACTA
Oligo 7	Phos-CGCTTAGTGCTATGATGCGTT/Digoxigenin-T/TCGCATCATAGCACTA
Oligo 8	AGGTCGCCGCCCAAAAAAAAAAAA-Digoxigenin
Oligo 9	Phos-CCTGGTGGGTCTCGATTCTCTT-Biotin
Oligo 10	Phos-GAGAATCGAGACCCA
Oligo 11	Phos-GCAAGCTTGCGGCCGCAC
Oligo 12	AGGTCGTTCGCTCCAAGC
crRNA 1	UUUCUUUUCACCAGUGAGACGUUUUAGAGCUAUGCU
crRNA 2	GCGUGCGCAGGCCGACGCACGUUUUAGAGCUAUGCU
tracrRNA	AGCAUAGCAAGUUAAAAUAAGGCUAGUCCGUUAUCAACUUGAAAAAGUGGCA CCGAGUCGGUGCUUU
EcUvrD for	TTCCCCTCTAGAAATAATTTTGTTTAACTTTAAGAAGGAGATATACATATGGACGT TTCTTACCTGCT
EcUvrD rev	GCCCTCGAGTTAACGCGACGGTGTGCAGAGCCCTCCATGATGGTGATGGTGAT GTGATGACACCGACTCCAGCCGGGCGT
EcExoI for	GGCAGCCATATGATGAATGACGGTAAGCA
EcExoI rev1	CCCTCCATGATGGTGATGGTGATGTGATGAGACAATCTCTTCCGCGTACTGCCA
EcExoI rev2	TATTTTGGATCCTTAACGCGACGGTGTGCAGAGCCCTCCATGATGGTGATGGTG ATGTGA
EcSSB for	GATATACATATGGCCAGCAGAGGCGTAAA
EcSSB rev	GAGCTCGAATTCTCAGAACGGAATGTCATCATCAA
EcSSB(A123C) for	GTCAGGGTGGTGGCGCTCCGTGCGGTGGCAATATCGGTGGTGG
EcSSB(A123C) rev	CCACCACCGATATTGCCACCGCACGGAGCGCCACCACCCTGAC

Supplementary Table 2. Mutation Rates in complementation assays

Strain	MMR proteins in expression plasmids*	Genotype	Mutation rates (10^{-8}) †	Mutation Range (10^{-8})
MG 1655	-	*wild type*	4.7	2.0-19.3
MG 1655 Δ*uvrD*	-	$uvrD^{-}$	327.8	219.1-736.3
MG 1655 Δ*uvrD*	EcUvrD-his_6-ald_6	$UvrD^{+}$	3.9	1.0-15.2

* Amino acid substitutions are indicated in parenthesis; the arrangement of the hexa-histidine (his_6) and Formylglycine Generating Enzyme (ald_6) recognition sequence relative to C-terminus (end) of the MMR gene.

† The median mutation rates are reported.

Supplementary Table 3. Excision Length Determined by Flow-stretching Experiment

[EcUvrD] nM	ssDNA Exonuclease	Nick	Exonuclease-dependent Excision Length (nt, mean ± s.e.)*
1	+ EcExoI	3'	184 ± 31
5	+ EcExoI	3'	90 ± 16
20	+ EcExoI	3'	45 ± 6
	+ EcExoI, EcRecJ, EcExoVII	3'	28 ± 12
	+ EcExoI, EcRecJ, EcExoVII	5'	50 ± 14
60	+ EcExoI	3'	45 ± 9
100	+ EcExoI	3'	48 ± 9

* Data were fit to a single exponential decay to derive the average length and corrected with background excision in the absence of ssDNA exonuclease (see **Methods**; **Supplementary Fig. 5b**).
